# Prevalence, Geographic Distribution, Risk Factors and Co-Infections of Feline Gammaherpesvirus Infections in Domestic Cats in Switzerland

**DOI:** 10.3390/v11080721

**Published:** 2019-08-06

**Authors:** Marilisa Novacco, Neda Ranjbar Kohan, Martina Stirn, Marina L. Meli, Adrian Alberto Díaz-Sánchez, Felicitas S. Boretti, Regina Hofmann-Lehmann

**Affiliations:** 1 Clinical Laboratory, Department for Clinical Diagnostics and Services, Vetsuisse Faculty, University of Zurich, 8057 Zurich, Switzerland; 2 Center for Clinical Studies, Vetsuisse Faculty, University of Zurich, 8057 Zurich, Switzerland; 3 Centro Nacional de Sanidad Agropecuaria (CENSA), San José de las Lajas 32700, Mayabeque, Cuba; 4 Clinic for Small Animal Internal Medicine, Vetsuisse Faculty, University of Zurich, 8057 Zurich, Switzerland

**Keywords:** cat, gammaherpesvirus, Switzerland, qPCR, virus load, hemoplasma, FeLV, FIV, retrovirus, veterinary sciences

## Abstract

Recently, a gammaherpesvirus was described in domestic cats (FcaGHV1). The goal of the present study was to investigate the presence of FcaGHV1 in Swiss domestic cats and analyze potential risk factors. Blood samples from 881 cats presented to veterinarians in all Swiss cantons and from 91 stray cats and neoplastic tissue samples from 17 cats with lymphoma were evaluated. FcaGHV1 was detected by real-time PCR targeting the glycoprotein B gene, followed by sequencing. Blood samples were also tested for feline hemoplasmas, feline leukemia virus (FeLV) and feline immunodeficiency virus (FIV). The molecular prevalence of FcaGHV1 was 6.0% (95% confidence interval (CI), 4.5–7.8%) in cats presented to veterinarians and 5.5% (95% CI, 1.8–12.4%) in stray cats. FcaGHV1 PCR-positive cats originated from 19/26 Swiss cantons. Factors significantly associated with FcaGHV1 detection included male sex, age >3 years, nonpedigree status and co-infection with FIV and hemoplasmas. Moreover, FeLV viremia tended to be associated with FcaGHV1 detection. High FcaGHV1 blood loads were found more frequently in FeLV-viremic cats and less frequently in hemoplasma-infected cats than in uninfected cats. Clinical information was unavailable for most of the 881 cats, but leukemia, carcinoma and cardiomyopathy were reported in FcaGHV1-positive cats. None of the tissue samples from the 17 cats with lymphoma tested positive for FcaGHV1. Sequence analyses revealed homogeneity among the Swiss isolates and >99.7% identity to published FcaGHV1 sequences. In conclusion, FcaGHV1 is present in Switzerland with a similar prevalence in cats presented to veterinarians and in stray cats. The pathogenic potential of FcaGHV1 needs further evaluation.

## 1. Introduction

Gammaherpesviruses (GHVs) are double-stranded DNA viruses. They belong to the Herpesviridae family, which includes three subfamilies: *Alpha*-, *Beta*- and *Gammaherpesvirinae* [1,2]. GHVs can infect humans, establishing a lifelong persistent infection mostly without evident clinical signs [3]. Host immunity is supposed to eliminate the infection, but frequently the virus undergoes latency with potential reactivation. GHV reactivation is suspected during co-infections or when cell-mediated immunity is compromised. In the latter cases, the virus can cause severe diseases that can be potentially fatal [4,5]. Two human GHVs are known to promote tumorigenesis in humans: Epstein–Barr virus and Kaposi’s sarcoma-associated herpesvirus [6,7,8,9]. Epstein–Barr virus is commonly identified in adult human beings worldwide. The virus is normally innocuous; however, in a few cases, it can cause lymphomas, carcinomas or other types of cancer. Loss of T-cell immunity and genetic predisposition are thought to be critical risk factors for the oncogenic potential of the Epstein–Barr virus [4,5].

GHVs are known to infect different mammalian species, and they are reported worldwide [10,11,12,13,14,15,16,17,18]. Novel GHVs were identified among Primates, Artiodactyla, Perissodactyla, Carnivora, Scandentia, and Eulipotyphla using PCR with panherpesvirus DNA polymerase gene primers or genus-specific glycoprotein B (gB) gene primers [19]. In 2014, the first gammaherpesvirus (named *Felis catus* gammaherpesvirus 1, FcaGHV1 [20]) was identified in domestic cats, followed by the identification of novel GHVs in other felids (bobcats, pumas, ocelots, leopard cats) in the USA and Japan [20,21,22]. Since that first identification, different epidemiological studies have shown that FcaGHV1 infection is widely endemic in domestic cats. Most epidemiological studies are based on the detection of the genus-specific glycoprotein B gene by PCR [19]. The reported worldwide prevalence of FcaGHV1 ranges from 1.3% to 23.6% in domestic cats [20,22,23,24,25,26,27]. Direct comparison between prevalence studies is difficult. Differences in prevalence may reflect variations in the studied cat population, the study inclusion criteria (such as feral free-roaming cats captured for neutering programs, feral cats housed in animal shelters or privately-owned cats) and the health status of the sampled cats. In addition, it is important to remember that the identification of FcaGHV1 DNA material does not provide information on the infection status of the animal and cannot differentiate between virus-infected cells, virion particles or free DNA [28]. More recently, a serological assay was developed to assess the exposure rate to FcaGHV1 within a cat population [29]. The seroprevalence of FcaGHV1 was found to be higher than the molecular prevalence detected by PCR [29], with approximately 50% of the FcaGHV1-seropositive cats being PCR-positive [20,27,29].

The pathogenic potential of FcaGHV1 in cats remains unclear. It has been shown that FcaGHV1 is more frequently identified in sick cats than in healthy cats [30]. In addition, age, sex, and concomitant co-infections have been identified as risk factors for FcaGHV1, with some regional variations. A significant association between FcaGHV1 and feline leukemia virus (FeLV) antigenemia was identified only in one study in Singapore [27], but this association was not confirmed recently [31]. The prevalence of FcaGHV1 is, however, increased in cats co-infected with feline immunodeficiency virus (FIV) and FeLV [27,31]. In addition, an association between FIV alone and FcaGHV1 has been reported in independent studies [23,25,26]. FIV is a widespread feline retrovirus sharing similarity with human immunodeficiency virus (HIV) that may cause an acquired immunodeficiency syndrome (AIDS)-like syndrome in infected cats. Additionally, FIV and HIV are two viral infections that can increase the risk of high-grade B cell lymphomas [30,32,33,34,35]. Interestingly, 15–20% of HIV-associated lymphomas are correlated with human gammaherpesviruses (Epstein–Barr virus and Kaposi’s sarcoma-associated herpesvirus) [32,36]. Thus, similar to human GHVs, FcaGHV1 in cats may be a cofactor for malignant transformation in lentivirus-infected individuals.

In the present study, we (i) assessed for the first time the molecular prevalence of FcaGHV1 in cats in Switzerland; (ii) evaluated potential risk factors associated with FcaGHV1 detection, such as geographic origin (canton), the age, sex and breed of the cat and co-infections with FeLV, FIV, and the three feline hemoplasma species; (iii) evaluated the presence of FcaGHV1 in neoplastic tissues of cats with lymphoma; and (iiii) sequenced a fragment (316 nucleotides) of the glycoprotein B gene of FcaGHV1 to further elucidate the phylogenetic relationship with other FcaGHV1 isolates and assess possible genetic variation in Switzerland.

Our study assessed the molecular prevalence of FcaGHV1 and risk factors associated with this infection in Switzerland for the first time. Remarkably, FcaGHV1 prevalence was similar in cats presented to veterinarians and in stray cats. In accordance with previous studies, factors significantly associated with FcaGHV1 detection included male sex, age >3 years, nonpedigree status and co-infections with FIV and hemoplasmas. Swiss FcaGHV1 isolates revealed homogeneity and >99.7% identity to previously published FcaGHV1 sequences.

## 2. Materials and Methods

### 2.1. Sample Collection

The study included two sets of feline blood samples. First, the study included 881 EDTA-anticoagulated blood samples collected from domestic cats presented to veterinarians in Switzerland between 2013 and 2016 [37]. The study was designed to include at least 20 blood samples from each of the 26 Swiss cantons, and samples were selected only by the postal code associated with the cat owners. The samples had been described and analyzed for FeLV provirus and antigen, and the results were previously presented elsewhere [37]. The samples were collected as part of a diagnostic workup by veterinarians for routine purposes, and only the remaining sample volume was used for this study. Therefore, no ethical approval was necessary to comply with Swiss regulations [38]. One cat per owner was included as far as known. In some cases, demographic data of the cats obtained at the time of sampling were available: breed (*n* = 639), sex (*n* = 641) and age (*n* = 636).

Second, 91 EDTA-anticoagulated blood samples collected from stray cats in the canton of Jura (Switzerland) were included. These samples were collected by veterinarians during a capture and neutering program for diagnostic purposes (health check). Demographic data of the cats were available in some cases: sex (*n* = 85) and age (*n* = 11). The samples had been collected in August and October 2014. No breed information was available for the cats. More results on these samples will be presented elsewhere [39].

In addition, lymphoma tissue samples from six field cats and 11 specific-pathogen-free (SPF) cats experimentally infected with FeLV and/or FIV were analyzed. Tissue samples from the six field cats were taken for routine diagnostic purposes only; therefore, no ethical approval was necessary to comply with Swiss regulations [38]. These samples included six lymph node, two liver, one spleen and two ileum samples. All of these latter cats were diagnosed with T-cell lymphomas, including one case of large granular lymphocyte lymphoma. All 11 SPF cats with lymphoma had been experimentally infected with FeLV in unrelated studies; two had additionally been infected with FIV [40,41,42,43,44,45,46,47,48]. At the start of the unrelated studies and before entering the SPF facility, all SPF cats had been tested for selected feline infectious agents, including alpha herpesvirus (feline herpesvirus 1) but not FcaGHV1 [49]. Tissue samples from the FeLV-infected SPF cats with high grade T-cell lymphoma (one with secondary blood infiltration) and from the FeLV-FIV-infected cats with mostly high grade T-cell lymphomas and one with high grade B-cell lymphoma had been collected during necropsy in unrelated studies [40,41,42,43,44,45,46,47,48]. Seven samples were from tumor tissues (including lymph node, thymus, and ovary), and four were bone marrow samples. All samples were snap-frozen in liquid nitrogen and stored at −80 °C until further use.

### 2.2. Nucleic Acid Extraction

Total nucleic acid (TNA) samples were extracted from 100 µL of EDTA-anticoagulated blood using a MagNaPure LC Total Nucleic Acid Isolation Kit (Roche Diagnostics, Rotkreuz, Switzerland) following the manufacturer’s instructions as reported previously [37]. All TNA samples were stored at −80 °C until further use. During all extractions, negative controls consisting of 100 µL of phosphate-buffered saline (PBS) were concurrently prepared with each batch of 15 samples to monitor for cross-contamination. For the analyzed tissues, samples were homogenized as previously described [50], and genomic DNA was extracted using a QIAamp DNA Tissue Kit (Qiagen, Hombrechtikon, Switzerland). Negative extraction controls consisting of phosphate-buffered saline were included with each batch.

### 2.3. Quantitative TaqMan Real-Time PCR Assays

To test for the presence of sufficient amounts of amplifiable TNA and the absence of significant PCR inhibitors, all TNA samples were analyzed for feline albumin (fALB) using real-time qPCR as previously described [51]. Samples with insufficient amplification (Ct value > 30) were re-extracted; detailed information was previously reported [37]. All samples with sufficient amounts of amplifiable TNA were tested for the presence of FcaGHV1 using a quantitative real-time qPCR assay targeting the viral glycoprotein B gene, as previously reported [20]. Each reaction was carried out in a final volume of 25 µL containing (final concentrations) 12.5 µL of 2× qPCR Mastermix (Eurogentec, Seraing, Belgium), 0.25 µL (0.5 U) of uracil-*N*-glycosylase (UNG, Eurogentec, Seraing, Belgium), 1.125 µL (900 nM) of each primer, 0.625 µL (250 nM) of probe, and 5 µl of template DNA. A synthetic double-stranded DNA (GeneArt String DNA, ThermoFisher Scientific Inc., Waltham, MA, USA) portion (600 bp) of the FcaGHV1 glycoprotein B gene containing the assay target sequence was used for assay validation and the quantification of viral loads. To determine the efficiency and linear range of amplification of the PCR assay, a 10-fold serial dilution of synthetic DNA template in 30 µg/mL carrier salmon sperm DNA (ThermoFisher Scientific, Reinach, Switzerland) spanning nine orders of magnitude (1 × 10^0^–1 × 10^8^ copies per reaction) was examined.

All samples had also been tested for FeLV proviral DNA, as previously reported [37]. Briefly, for the detection of FeLV provirus, a real-time qPCR assay specific for exogenous FeLV LTR U3 was applied [45].

Moreover, 106 of the 881 samples were additionally tested for the three feline hemoplasmas: *Mycoplasma haemofelis* (Mhf), ‘*Candidatus* Mycoplasma haemominutum’ (CMhm) and ‘*Candidatus* Mycoplasma turicensis’ (CMt). The 106 samples included 53 FcaGHV1 PCR-positive samples and 53 FcaGHV1 PCR-negative samples. The 53 FcaGHV1 PCR-negative samples were chosen from all the FcaGHV1 PCR-negative samples to match—as well as possible—the FcaGHV1 PCR-positive samples concerning the following parameters: the origin (Swiss canton of provenience), age, sex and pedigree status of the cat. There was no significant difference between FcaGHV1 PCR-negative and positive cats regarding sex (pChi2 = 0.9171), percentage of pedigree cats (pChi2 = 1.000) and age (pChi2 = 0.0794) (Supplementary Figure S1). The three feline hemoplasmas (Mhf, CMhm, and CMt) were detected, and blood loads were quantified using specific real-time qPCR assays targeting the 16S rRNA gene as previously described [52,53]. The real-time qPCR detection limit for the hemoplasmas (all three assays) is one copy per PCR, which corresponds to 200 copies/mL of blood [52,53].

In addition, lymphoma tissue samples from six field cats and 11 specific-pathogen-free (SPF) cats experimentally infected with FeLV and/or FIV were tested for FeLV proviral DNA [37] and FIV proviral DNA [54]. For FIV, a real-time RT-PCR was performed according to a modified version of the method used by Wang et al. [55], which used 900 nM of both the upstream (FIV_gag_upstr: 5′- ATG GGG AAY GGA CAG GGG CGA GA-3′) and downstream (FIV_gag_downstr: 5′- TCT GGT ATR TCA CCA GGT TCT CGT CCT GTA-3′) primers and 250 nM fluorogenic probe (FIV_gag_F2ABCEmIM 5′-FAM-TGG CCA TWA ARA (iQ500)GAT GYA GTA ATG TTG CTG TAG G-BHQ1-3′), 0.625 µL (40 U/µL) RNasin^R^ Plus (Promega, Madison, WI, USA), 12.5 µL 2× RT qPCR Reaction Mix, 0.5 µL Superscript^TM^ III RT/Platinum^R^ Taq mixture and 0.05 µL ROX (all from the Superscript^TM^ III Platinum^TM^ One-Step qRT-PCR kit, ThermoFisher Scientific, Carlsbad, CA, USA) in a final volume of 25 µL. The reaction mix underwent reverse transcription at 50 °C for 15 min, denaturation and activation for 2 min at 95 °C and 45 cycles of 95 °C for 15 s and 60 °C for 30 s.

All qPCR assays were performed using an ABI 7500 Fast Sequence Detection System (Applied Biosystems, ThermoFisher Scientific, Reinach, Switzerland). Positive and negative controls were included in each PCR run. The primers and probes used in the present study are shown in Table 1.

### 2.4. Detection of FeLV Antigen and FIV Antibodies

Among the 881 blood samples, FeLV provirus-positive samples were additionally tested for FeLV p27 antigen [37] using a sandwich enzyme-linked immunosorbent assay [56]; antigenemia is a marker of FeLV infection and, in most but not all cats, a parameter of viremia [57]. Cats were considered antigenemic when the antigen level in their samples was 20% of the positive control value in the p27 antigen ELISA [37]. Of the 106 matched samples (see above), 104 were also tested for FIV. For two of the 106 matched samples, no material remained for FIV testing; these samples included one FcaGHV1-positive and one FCaGHV1-negative sample. Antibodies against FIV were detected by Western blot analysis as described previously [58,59]. Samples with antibodies against the p15 and p24 bands were considered positive.

### 2.5. Sequencing and Phylogenetic Analysis of the FcaGHV1 Swiss Isolates

Three FcaGHV1-positive samples were selected and subjected to sequencing. For this purpose, a 360 bp fragment of the glycoprotein B gene was amplified by PCR (Table 1) as previously described [27]. The reaction was performed in a T-personal 48 thermal cycler (Biometra GmbH, Göttingen, Germany) in a total volume of 50 µL containing 10 µL of 5× Phusion HF buffer (Finnzymes, Espoo, Finland), 500 nM each primer, 200 nM each deoxynucleoside triphosphate (dNTP) (Sigma-Aldrich, Buchs, Switzerland), 1 U Phusion DNA Polymerase (Finnzymes, Espoo, Finland) and 5 µL of template DNA. The thermal cycling program comprised a step at 98 °C for 3 min; 35 cycles at 98 °C for 10 s, 57 °C for 30 s, and 72 °C for 30 s; and a final step at 72 °C for 10 min.

PCR products were analyzed on a 1.5% agarose gel prestained with a commercial gel stain (GelRed™ Nucleic Acid Stain, Biotium, Inc., Fremont, CA, USA) and were then visualized by UV transillumination. The AccuBand™ 100 bp DNA ladder II (SMOBIO^®^, Hsinchu City, Taiwan) was used as the standard to initially determine the molecular mass of PCR products. Amplicons with the expected size were excised from the gel, purified using a QIAquick Gel Extraction Kit (Qiagen AGHombrechtikon, Switzerland), and cloned using a TOPO TA Cloning Kit with the PCR™II-TOPO^®^ vector and One Shot TOP10 chemically competent *Escherichia coli*, based on the manufacturer’s instructions (Invitrogen, Basel, Switzerland). The recombinants obtained were selected and grown in large quantities for plasmid extraction using a QIAprep Spin Miniprep Kit (Qiagen AG, Hombrechtikon, Switzerland) and were subjected to automated DNA sequencing with vector-specific M13 forward and reverse primers at a commercial laboratory (Microsynth, Balgach, Switzerland) under standard conditions. Sequences were edited and assembled using Geneious^®^ (v 11.1.5, Biomatters Limited, Auckland, New Zealand). After trimming the vector and primer sequences, the resulting 316 bp consensus sequence of the glycoprotein B gene was aligned and compared for similarity with those available in the GenBank database using Basic Local Alignment Search Tool (BLAST) analysis [60].

Phylogenetic analysis was conducted using MEGA-X (Molecular Evolutionary Genetics Analysis, Kumar) [61]. Sequences were aligned to additional reference sequences retrieved from GenBank using the ClustalW algorithm [62]. The phylogenetic tree was inferred via the maximum likelihood method using a distance matrix corrected for nucleotide substitutions based on the Kimura 2-parameter model [63]. The dataset was resampled 1000 times to generate bootstrap values [64]. The partial FcaGHV1 glycoprotein B nucleotide sequences obtained in this study have been deposited in the GenBank database under accession numbers MK572745–MK572747.

### 2.6. Statistical Analysis

Statistical analysis was performed using GraphPad Prism (version 8.1, GraphPad Software, San Diego, CA, USA). The FcaGHV1 molecular prevalence rates were calculated with 95% confidence intervals (CI). Frequencies were compared using Fisher’s exact test for small numbers (pF) and using the chi-square test (pChi2). To compare continuous variables (FcaGHV1 blood loads) between two groups, the nonparametric Mann–Whitney U-test was used (pMW). The following variables were tested for statistical association with FcaGHV1 detection in the first set of 881 cats presented to veterinarians: origin (canton of Switzerland), breed (pedigree and nonpedigree cats), age (≤3 years vs. >3 years), sex and reproductive status (castrated male, intact male, spayed female, and intact female), FeLV provirus and antigen positivity, and FIV and hemoplasma infection status. Considering the small number of cats with sufficient data in the second set of samples (*n* = 91 stray cats from the canton of Jura), no statistical analyses were performed concerning the age and sex of the cats. Correlations between the FcaGHV1 blood loads and ages of the cats and the FcaGHV1 molecular prevalence and human population density was calculated using nonparametric Spearman rank correlation (pS; *r* = correlation coefficient). *P*-values < 0.05 were considered significant.

## 3. Results

### 3.1. Efficiency, Sensitivity and Linear Range of Amplification of the FcaGHV1 Real-Time qPCR Assay

**Figure 1:**
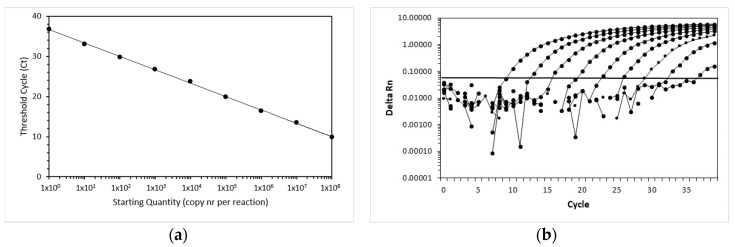
Linear range of amplification for the domestic cat gammaherpesvirus (FcaGHV1) real-time qPCR assay. (**a**) Amplification plots of a representative real-time qPCR assay show the cycle number versus the normalized reporter dye fluorescence. (**b**) The standard curve of a representative real-time qPCR assay shows the starting input quantity (copies per reaction) in a 10-fold serial dilution of standard template versus the measured threshold cycle. Rn is the fluorescence of the reporter dye divided by the fluorescence of a passive reference dye. DeltaRn is the normalized value of Rn obtained by subtracting the baseline value.

The efficiency and linear range of amplification of the FcaGHV1 real-time qPCR assay were determined using 10-fold serial dilutions of the standard. Amplification of FcaGHV1 showed linearity over the whole range of dilution (Figure 1). The highest dilution still yielded a positive signal containing one copy per reaction. In the endpoint dilution experiment, the dilution with 1 copy yielded 8 positive reactions of 10. For the next lower dilution, none of the 8 reactions was positive. Thus, the lower limit of detection was 1 copy of template per 5 µL reaction, corresponding to 200 copies per milliliter of blood.

### 3.2. Sample Characteristics

In the first set of 881 samples from cats presented to veterinarians throughout all cantons of Switzerland (Table 2), the samples originated from 160 pedigree and 479 nonpedigree cats, and no information was available for 242 samples (Table 3). The sex and reproductive status data revealed 308 castrated male and 207 spayed female cats and 56 male and 70 female intact animals (the sex and reproductive status of 240 cats were unknown). The cats’ ages varied from 2 months to 21.5 years; the median age was 6.6 years (the age of 244 cats was unknown).

For the second set of samples collected from 91 stray cats in the canton of Jura, limited information was available: 48 cats were intact females and 37 cats were intact males; the information was unavailable for six cats. The ages of the 11 cats with available information were <1 year for five cats, 1–3 years for four cats and >3 years for four cats.

### 3.3. Sample Prevalence of FcaGHV1 in Swiss Domestic Cats

**Figure 2:**
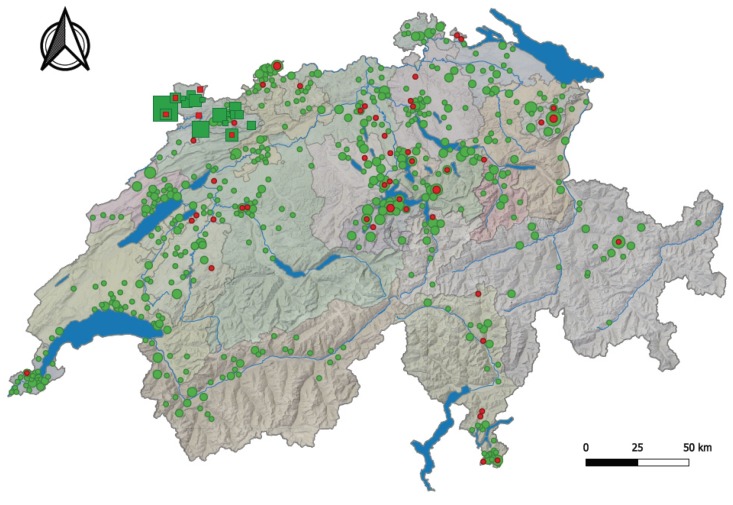
Origin of the 881 cats presented at veterinary facilities in all 26 cantons of Switzerland and of the 91 stray cats from Jura. FcaGHV1 PCR-positive samples from cats presented to veterinarians are represented by red circles, and those from stray cats in Jura by red squares. FcaGHV1 PCR-negative samples from cats presented to veterinarians are shown as green circles, and those of stray cats as green squares. The circle diameters are proportional to the number of positive or negative cases in the considered village/town. The squares and circles are not directly proportional; the squares are somewhat reduced in size for better visualization. The Swiss cantons are shaded in different colors. Major lakes and rivers are depicted in blue.

Of the 881 cats from all cantons of Switzerland, 53 tested as FcaGHV1-positive (6.0%; 95% CI, 4.5–7.8%). FcaGHV1 PCR-positive samples originated from 19 of the 26 Swiss cantons (Table 2 and Figure 2). Overall, although the molecular prevalence ranged from 0% to 16.7% in each canton, there was no significant difference in FcaGHV1 molecular prevalence among the different cantons of Switzerland (pChi2 = 0.1576; Table 2; Figure 2). From Figure 2, however, it is evident that fewer FcaGHV1-positive cats were found in the Alps than in the other two major Swiss natural environments—the Swiss Plateau and the Jura region. The highest molecular prevalence of FcaGHV1 was found in the canton of Jura (16.7%; 95% CI, 4.7–37.4%), followed by the canton of Appenzell Innerrhoden (12.5%; 95% CI, 2.7–32.4%) and the canton of Ticino (12.0%; 95% CI, 4.5–24.3%; Table 2).

We aimed to assess whether the density of the cat population in the different Swiss cantons plays a role in FcaGHV1 molecular prevalence. Since no data are available on the cat density in each Swiss canton, we used the human density as an approximation [65]. The human population density in the three cantons showing the highest FcaGHV1 molecular prevalence (Jura, Appenzell Innerrhoden and Ticino) ranges from 87 to 129 people/km^2^ [65]. FcaGHV1 was not detected in any of the tested cats from seven cantons: Glarus, Solothurn, Neuchâtel, Sant Gallen, Thurgau, Vaud and Valais. The human population density in these cantons ranges from 59 to 341 people/km^2^ [65]. There was no statistical association between the human population density and the FcaGHV1 molecular prevalence in cats (*n* = 26; pS = 0.4649; *r* = 0.01818; 95% CI, −0.3822–0.4128).

Among the stray cats originating from the canton of Jura, five of 91 were FcaGHV1-positive (5.5%; 95% CI, 1.8–12.4%). There was no significant difference in the molecular prevalence of FcaGHV1 between stray cats (5.5%; 95% CI, 1.8–12.4%) and cats presented to veterinarians in the canton of Jura (4 of 24; 16.7%; 95% CI, 4.7–37.4%; pF = 0.4664).

### 3.4. Association of FcaGHV1 Detection with Nonpedigree Status, Male Sex and Age >3 Years

The association of FcaGHV1 detection with breed, sex and age was assessed in the 881 cats presented to veterinarians. Pedigree cats were significantly less frequently FcaGHV1-positive (2/160; 1.3%; 95% CI, 0.2–4.4%) than nonpedigree cats (39/479; 8.1%; 95% CI, 6.0–10.9%; pF = 0.0012; odds ratio (OR) 7.0; 95% CI, 1.9–29.8; Table 3). The two pedigree cats that were FcaGHV1-positive were one Sphinx and one Maine Coon cat. There was a significant difference in sex and reproductive status between FcaGHV1-positive and negative cats (pChi2 = 0.0223; Table 3). Male cats (intact and castrated) were more frequently FcaGHV1-infected (33/364; 9.1%; 95% CI, 6.5–12.5%) than female cats (intact and spayed; 10/277; 3.6%; 95% CI, 2.0–6.5%; pF = 0.0064; OR 2.7; 95% CI, 1.3–5.4). There was a significant difference in the age distribution between FcaGHV1-positive and negative cats (pChi2 = 0.0422; Table 3). When the molecular prevalence in the different age groups was examined, it became evident and was confirmed by statistical analysis that cats older than 3 years were significantly more frequently FcaGHV1-positive (39/440; 8.9%; 95% CI, 6.6–11.9%) than cats less than 3 years of age (3/196; 1.5%; 95% CI, 0.4–4.4%); pF = 0.0002; OR 6.3; 95% CI, 2.0–19.5).

### 3.5. Association of FcaGHV1 with Other Infectious Agents

In the first set of feline samples (*n* = 881), 47 samples were FeLV provirus-positive (5.3%; 95% CI, 3.9–7.0%), of which four were also FcaGHV1-positive (Table 4); in addition, 18 samples were viremic (2.0%; 95% CI, 1.2–3.2%), of which three were also FcaGHV1-positive (Table 4). FeLV-viremic (p27 antigen-positive) cats tended to be more frequently FcaGHV1-positive (3 of 18; 16.7%) than FeLV-aviremic cats (50 of 863; 5.8%; pF = 0.0884; OR 3.3; 95% CI, 0.97–11.1; Table 4). There was no statistical difference between the molecular prevalence of FcaGHV1 in FeLV provirus-positive cats and that in FeLV provirus-negative cats (pF = 0.5202; Table 4).

In addition, 104 of the 881 samples were tested for FIV, including 52 FcaGHV1-positive samples and 52 of the FcaGHV1-negative matched samples. Among the 104 tested samples, 23 were positive for FIV by Western blot analysis (22.1%; 95% CI, 14.6–31.3%). FIV-positive cats were more frequently FcaGHV1-positive (20 of 23; 87.0%) than FIV-negative cats (32 of 81; 39.5%; pF <0.0001; OR 10.2; 95% CI, 2.8–37.2; Table 5). Overall, 106 of the 881 samples were also tested for feline hemoplasma infections, including the 53 FcaGHV1-positive and 53 matched FcaGHV1-negative samples. Among these 106 samples, 27 were positive for at least one hemoplasma species (25.5%; 95% CI, 17.5–34.9%). Cats positive for any feline hemoplasma species were significantly more frequently FcaGHV1-positive (19 of 28; 67.9%) than hemoplasma-negative cats (34 of 78; 43.6%; pF = 0.0463; OR 3.1; 95% CI, 1.2–8.0; Table 5). The same pattern held for CMhm-positive cats, since all hemoplasma-positive cats were also positive for CMhm: cats positive for CMhm were significantly more frequently FCaGHV1-positive than CMhm-negative cats (pF = 0.0463; OR 3.1; 95% CI, 1.2–8.0). None of the cats had a single infection with Mhf or CMt. Of the 19 cats positive for both hemoplasma and FcaGHV1, four were concurrently PCR-positive for more than one hemoplasma species, including three cats co-infected with CMt and CMhm and one cat co-infected with CMhm and Mhf. All the FcaGHV1-negative cats were PCR-positive only for CMhm. None of the FcaGHV1-negative cats were PCR-positive for more than one feline hemoplasma. No blood samples of the cats presented to veterinarians tested positive for all three hemoplasma species.

In the second set of feline samples (*n* = 91 stray cats from the canton of Jura), 7 samples were FeLV provirus-positive (7.7%; 95% CI, 3.1–15.2%), but none of the FeLV provirus-positive cats were also FcaGHV1 PCR-positive. No serum samples were available to test for FIV. Seventeen of the 91 whole blood samples were positive for at least one hemoplasma species (18.7%; 95% CI, 11.3-28.2%). Notably, all five FcaGHV1-positive cats were also hemoplasma-positive (Table 6), and hemoplasma PCR-positive cats were more frequently FcaGHV1-positive (5 of 17; 29.4%) than FcaGHV1-negative (0 of 74; 0%; pF = 0.0001; OR 65.6; 95% CI 3.4–1261). Among the 91 tested samples, seven tested PCR-positive for Mhf (7.7%; 95% CI, 3.1-15.2%), 12 for CMhm (13.1%; 95% CI, 7.0-22%), and two for CMt (2.2%; CI, 0.3-7.7%). Among those samples, two were concurrently PCR-positive for all three hemoplasma species (Mhf, CMhm, and CMt). One of these two samples was also FcaGHV1 PCR-positive.

### 3.6. FcaGHV1 Blood Loads

The FcaGHV1 blood loads in this study ranged from 200 (the detection limit) to 3.9 × 10^5^ copies/mL of blood. There was no significant difference in the blood loads between the samples from the 53 FcaGHV1-positive cats presented to veterinarians and the five FcaGHV1-positive stray cats (pMW = 0.4956). When the FcaGHV1 blood loads in the 881 cats presented to veterinarians were grouped into “low” (<800 copies/mL; *n* = 31) and “high” loads (>1000 copies/mL; *n* = 22), FeLV provirus-positive cats significantly more frequently exhibited high FcaGHV1 loads (4/4; 100%; 95% CI, 40–100%) than FeLV provirus-negative cats (18/49; 37%; 95% CI, 23–52%; pF = 0.0250; OR 15.3; 95% CI, 0.8–301). There was also a tendency for FeLV-viremic cats to more frequently exhibit high FcaGHV1 loads (3/3; 100%; 95% CI, 29–100%) than FeLV-aviremic cats (19/50; 38%; 95% CI, 25–53%; pF = 0.0657; OR 11.3; 95% CI 0.6–231). In contrast, hemoplasma-positive cats significantly less frequently exhibited high FcaGHV1 loads (4/19; 21%; 95% CI, 6.1–46%) than hemoplasma-negative cats (18/34; 52%; 95% CI, 35–70%; pF = 0.0408; OR 0.23; 95% CI, 0.07–0.86). There was no statistical difference in the FcaGHV1 loads between FIV-positive and FIV-negative cats (pMW = 0.1295). In addition, there was no statistical association between the FcaGHV1 blood loads and the age of the positive cats (*n* = 42; pS = 0.5495; r = 0.095; 95% CI, -0.224–0.396).

### 3.7. Clinical Presentation of Some FcaGHV1-Positive Cats

Clinical information (presenting clinical signs, pretreatment, and diagnosis) was available for selected FcaGHV1 PCR-positive cats presented to the Clinic for Small Animal Internal Medicine (*n* = 7). This information was compared to that of age-, sex- and breed-matched FcaGHV1 PCR-negative cats presented at the same clinic in this study (*n* = 12).

Four of the seven FcaGHV1-positive cats had a history of pretreatment with glucocorticoids for one or several days, and only two were current on their vaccinations against feline parvovirus, feline herpesvirus-1 and feline calicivirus. Five of the seven FcaGHV1-positive cats had cardiorespiratory signs, with final diagnoses of hypertrophic cardiomyopathy (HCM) (*n* = 3) and/or bronchopneumonia (*n* = 2), pyothorax (*n* = 1), and feline asthma (*n* = 1). In the latter cat, squamous cell carcinoma of the cheek with metastasis to the regional lymph node was confirmed after surgical excision. In one of the two cats without cardiorespiratory signs (cat 2223639), immune-mediated hemolytic anemia was diagnosed, which, after 4 weeks of glucocorticoid treatment, was determined to be acute leukemia. Further characterization of the acute leukemia was not possible. The other positive cat (cat 2236869) was 20 years old with chronic kidney disease (CKD) and secondary chronic vomiting and weight loss.

In comparison, only 2 of the 12 FcaGHV1-negative cats with clinical information had a history of pretreatment with glucocorticoids (one day), and all except one were current on their vaccinations. None of those cats showed cardiorespiratory signs; 7/12 presented because of gastrointestinal signs (vomiting, diarrhea and/or anorexia). The final diagnoses in these cats were pancreatitis (*n* = 2), food-responsive inflammatory bowel disease (*n* = 1), cholangitis (*n* = 1), GI bleeding (*n* = 1) and lymphoma (*n* = 2). Three of the other 5 cats showed neurological signs (permethrin intoxication, *n* = 1; otitis media, *n* = 1; meningioma, *n* = 1). The final diagnoses in 2 of the 5 cats were diabetes mellitus and fibrosarcoma (showing a large mass on the lateral abdomen). There was no difference in outdoor access between the FcaGHV1 PCR-positive and -negative cats. The clinical diagnoses of the seven selected FcaGHV1-positive cats are shown in Table 7.

### 3.8. Sequence Analysis

**Figure 3:**
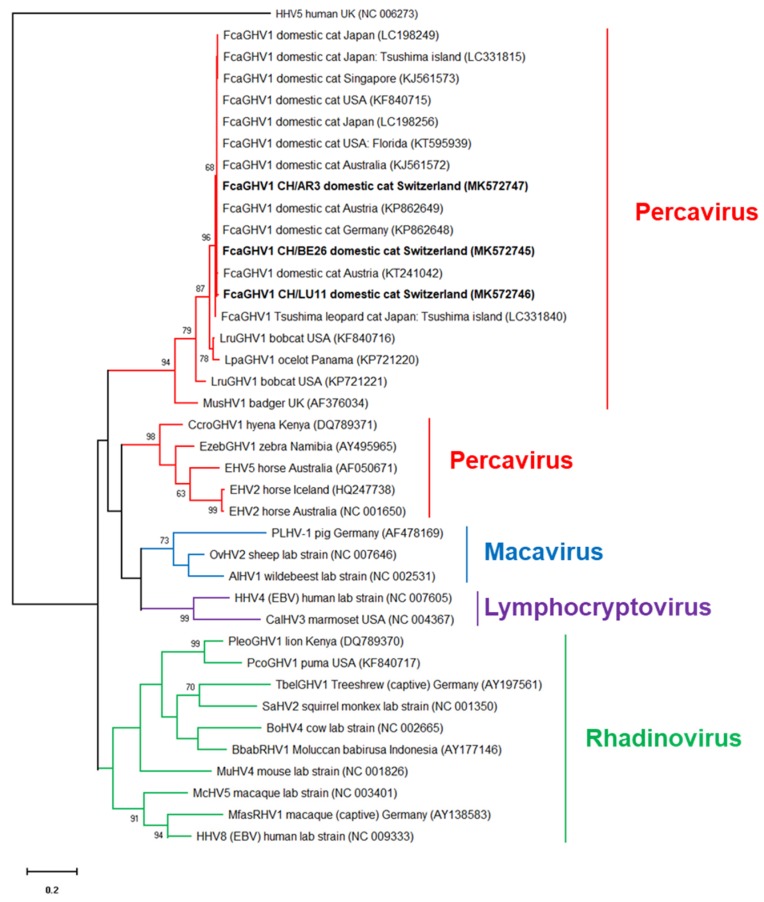
Molecular phylogenetic analysis by the maximum likelihood method of gammaherpesviruses using the glycoprotein B gene. The evolutionary history was inferred using the maximum likelihood method based on the Kimura 2-parameter model [63]. The tree with the highest log likelihood (-6535.03) is shown. The percentage of trees in which the associated taxa clustered together is shown next to the branches. The gammaherpesvirus genera (Percavirus, Macavirus, Lymphocryptovirus and Rhadinovirus) are shown in color. Initial tree(s) for the heuristic search were obtained automatically by applying the Neighbor-Join and BioNJ algorithms to a matrix of pairwise distances estimated using the maximum composite likelihood (MCL) approach and then selecting the topology with the highest log likelihood value. The tree is drawn to scale, with branch lengths measured in the number of substitutions per site. Bootstrap support from 1000 replicates is shown for each branch node (values below 60 are not displayed). The analysis included 39 nucleotide sequences. There was a total of 335 positions in the final dataset. Evolutionary analyses were conducted in MEGA X [61]. The betaherpesvirus human cytomegalovirus (HHV5) was the outgroup.

The results of the FcaGHV1 qPCR assay were confirmed by the sequencing of a 316 bp fragment of the glycoprotein B gene of FcaGHV1 from three random samples. Two samples (BE26 and AR3) showed identical sequences, and the third sample (LZ11) differed from the others by one nucleotide (a synonymous nucleotide polymorphism). The sequences of the former samples were 100% identical to the FcaGHV1 sequence from Australian infected cats (GenBank KJ561572 [27]) and sequences from Germany, Austria, the USA and Japan (KP862648, KP862649, KF840717, and LC198234, respectively [20,23,25]) and 99.7% identical to that from a Singaporean cat (KJ561573 [27]). Phylogenetic analysis showed that the Swiss FcaGHV1 sequences (MK572745-MK572747) clustered together with FcaGHV1 sequences from domestic cats, bobcats and ocelots, in the genus Percavirus (Figure 3).

### 3.9. Absence of FcaGHV1 in Lymphomas

None of the analyzed tumor or bone marrow samples from the 17 cats with lymphoma were positive for FcaGHV1. This sample set included samples from nine SPF cats experimentally infected with FeLV, two cats coinfected with FeLV and FIV, and six samples collected from clinical cases in field cats. The latter six samples collected from the clinical cases tested PCR-negative for FeLV and FIV. The lymphoma cases included both B- and T-cell lymphoma.

## 4. Discussion

This study is the first to investigate the presence of FcaGHV1 in Switzerland. A molecular prevalence of 6.0% (95% CI, 4.5–7.8%) was found for FcaGHV1 in domestic cats presented to veterinarians throughout Switzerland. A similar molecular prevalence was noted in stray cats in Switzerland (5.5%; 95% CI, 1.8–12.4%). FcaGHV1 PCR-positive cats originated from 19 of the 26 Swiss cantons, and no significant difference in the prevalence of FcaGHV1 infection was observed among the different cantons. However, there was an obvious geographic clustering of FcaGHV1 detection in the Swiss Plateau and Jura region of Switzerland compared to the Alps. This pattern could indicate that the climate or certain vectors could play a role in FcaGHV1 transmission. We also investigated whether there was an association with population density; as no data are available on the cat density in each Swiss canton, we used the human density as an approximation [65], assuming that a higher number of people indicates a higher number of cats. There was no association between the density of the human population and the molecular prevalence of FcaGHV1 infection in domestic cats. Thus, either our assumption was wrong and many of the cats in more highly populated regions do not have outdoor access providing contact to other cats, or cat density is not associated with the FcaGHV1 detection.

In contrast, we did find an association between FcaGHV1 detection and the sex of the cats. Male cats were more likely to be infected with FcaGHV1 than female cats. A higher FcaGHV1 infection risk for male cats was reported in prevalence studies from the USA, Europe, Brazil, Tsushima Island (Japan) and Australia [20,22,23,24,26,27]. Male cats were also more likely to be seropositive than female cats, using the newly developed serological assay for FcaGHV1 [29]. It has been speculated that male cats may be at higher risk of viral exposure due to their strong territorial behavior, which often leads to aggressive interactions among cats [20,23,27,28]. Biological factors such as hormonal differences between male and female cats could also potentially influence host immunity or viral replication [20,66]. Cross-sectional epidemiological data using structural equation models further showed that host phenotypic traits such as aggressive male phenotypes are real FcaGHV1 disease risk factors and not simply correlated events [67]. This important epidemiological finding suggests horizontal transmission by direct animal contact (e.g., during territorial aggression or through contaminated objects) as one possible route of FcaGHV1 infection. This hypothesis is supported by the recent identification of FcaGHV1 in oronasal swabs and tissues collected from infected cats [68]. Oronasal secretions, therefore, might play an important role in the transmission of FcaGHV1 among cats.

We found that cats greater than three years of age were at increased risk of being FcaGHV1 PCR-positive. The age of the cats also seems to play a role in the FcaGHV1 prevalence in other studies, where adult cats were more frequently infected than young cats [20,23,25,26,27,29]. The observation that adult cats are more frequently infected than young animals supports the assumption that FcaGHV1 infection cannot be cleared by the cat’s immune system, leading to lifelong infection and increasing infection rates with increasing age of the cats. Interestingly, it was recently reported that cats can be infected with FcaGHV1 from two months of age [68]. None of the younger cats (< 2 months of age) were FcaGHV1 PCR-positive [68].

FcaGHV1 infections have been associated with several other infectious agents [23,24,25,26,27]. In the present study, FIV was identified as a risk factor for FcaGHV1 detection. FIV was previously recognized as a risk factor, and higher FcaGHV1 blood loads were found in FIV-infected cats than in FIV-negative cats in Europe, Australia [23,27], Brazil [26] and Japan [25]. The association between FcaGHV1 and FIV may be because FIV may lead to immunosuppression in infected cats, and this, in turn, may facilitate FcaGHV1 replication or escape from latency [27]. FeLV viremia also tended to be associated with FcaGHV1 detection. We found a tendency for FeLV viremic cats to be more frequently FcaGHV1-infected than FeLV aviremic cats. Progressive FeLV infection with persistent FeLV viremia was previously reported to induce loss of cell (neutrophil and lymphocyte) function and changes in cytokine patterns, leading to the clear dysfunction of the immune system and a decrease in tumor surveillance mechanisms, causing an increased risk of tumor development in infected cats [69]. These alterations of the immune system in viremic FeLV-infected cats may explain the tendency toward the increased frequency of FcaGHV1 detection in FeLV-viremic cats compared to that in FeLV-aviremic cats. Interestingly, we also found that FeLV-viremic and FeLV provirus-positive cats more frequently exhibited high FcaGHV1 blood loads than uninfected cats. FeLV was not consistently identified as a risk factor for FcaGHV1 alone [27,31], but cats co-infected with FIV and FeLV were identified as having an increased risk [31]. It is known that FeLV–FIV co-infections have a more severe influence on a cat’s immune system than FIV or FeLV alone [43,69,70]. The present study showed that both single FIV and single FeLV infection may have an effect on FcaGHV1 detection. It cannot be determined whether this effect is actually due to an immunosuppressive effect of feline retroviruses, since immunological parameters such as CD4+ counts have not been investigated in FcaGHV1-infected cats, or whether the association could also be based on similar transmission routes. However, in stray cats, no association was found between FeLV infection and FcaGHV1 detection; this lack of association could imply that not a similar method of transmission but rather actual immunosuppression, which is more detrimental and probably more quickly fatal in stray cats than in privately-owned cats presented to veterinarians, is the basis of this association. Alternatively, the small number of stray cats may have limited the statistical analysis; however, this possibility seems less likely since the percentage of FeLV provirus-positive stray cats (7.7%) was rather high and was higher than that of cats presented to veterinarians (5.3%).

We also identified hemoplasma infections as risk factors for FcaGHV1 in cats presented to veterinarians as well as in stray cats. Hemoplasmas were also previously recognized as risk factors for FcaGHV1 [24,27]. Again, the real meaning of the association between hemoplasma and FcaGHV1 infections has not yet been identified; however, these two infections may indeed share similar transmission routes, such as aggressive contact between cats in an outdoor environment or vector-borne transmission. In addition, we found two cats concurrently infected with all three feline hemoplasma species: Mhf, CMhm and CMt. Both cats were stray cats, and one of the two cats was also FcaGHV1 PCR-positive. Concurrent infections with all three feline hemoplasmas have rarely been reported previously.

The pathogenic potential of FcaGHV1 in domestic cats is not completely understood. It was shown that FcaGHV1 PCR-positive cats are 2.8 times more likely to be ill than healthy [27]. However, this association (FcaGHV1 and illness) could not be shown in other studies [23]. In the present study, clinical information was available only for a limited number of cats; none of the FcaGHV1-positive cats had lymphoma, but two of the matched FcaGHV1-negative cats did. This finding was consistent with the negative results of all our tested lymphoma tissue samples. Interestingly, the majority of the FcaGHV1-positive cats with available clinical data showed cardiorespiratory signs, with a confirmed diagnosis of hypertrophic cardiomyopathy in three of these cats. Although one of these cats was a Maine Coon cat, a breed in which the incidence of hypertrophic cardiomyopathy is known to be increased, none of the three FcaGHV1-negative Maine Coon cats exhibited hypertrophic cardiomyopathy. An association between FcaGHV1 and cardiomyopathy has not been reported previously. However, Epstein–Barr virus, another GHV, was detected in humans with cardiomyopathy, suggesting a possible association between the virus and the otherwise unexplained cardiomyopathy [71]. FcaGHV1 DNA was detected in multiple tissues from infected cats, including heart tissue [27]. More studies are needed to further evaluate the possible association of FcaGHV1 and cardiomyopathy in a larger number of cats. Whether glucocorticoid or immunosuppressive treatment and vaccination status have any influence on FcaGHV1 infection and pathogenesis can only be speculated, as our number of cases was small.

In humans, two GHVs (Epstein–Barr virus and Kaposi’s sarcoma-associated herpesvirus) are associated with the development of cancer [72]. FcaGHV1 was isolated in T and B lymphocytes of cats but not in feline myeloid cells [73]. This pattern supports the hypothesis that FcaGHV1 is lymphotropic, as reported for the other GHVs. In a recent study analyzing 122 feline lymphoma cases (including histological and/or cytological preparations), no association was found between FcaGHV1 and the development of lymphoma in cats [74]. The absence of an association, however, does not rule out a pathogenic role of the virus [74]. To further evaluate the role of FcaGHV1 in lymphomagenesis, transcriptome analysis and in situ hybridization were performed on tissue samples of cats with lymphoma [75]. In that study, FcaGHV1 was detected in some FIV-associated lymphomas, and a positive FcaGHV1 intranuclear signal was visible by in situ hybridization in >90% of the cells in a case of feline large granular lymphocyte lymphoma. These results demonstrated that FcaGHV1 was present in feline lymphoma but only at low copy numbers, impairing the possibility of evaluating the role of the virus in lymphomagenesis [75]. High FcaGHV1 tissue loads (48 FcaGHV1 genomes per cell) were previously detected in a lymph node from a cat with high-grade T-cell intestinal lymphoma [76], suggesting a certain variability of the viral loads within feline lymphoma subtypes. Further studies investigating the cellular location and expression of FcaGHV1 antigens within neoplasias are needed to clarify this possibility. In the present study, all tissue samples from cats with lymphoma—six clinical cases testing negative for FeLV and FIV and 11 cats experimentally infected with FeLV and/or FIV—were FcaGHV1-negative. One of the cats also had large granular lymphocyte lymphoma. It is important to mention that the lack of association between FcaGHV1 and the development of lymphoma seen in the present study does not rule out a role of FcaGHV1 in lymphomagenesis, especially considering the inclusion of the tissue samples from the 11 SPF-cats that had been kept in a research facility under barrier conditions. However, when the cats had been purchased from an accredited SPF breeding facility some years ago, FcaGHV1 had not yet been discovered and no screening assays were available to detect the virus. Thus, FcaGHV1 was not on the list of specified pathogens yet, and the cats had not been tested for FcaGHV1 prior to the FeLV and/or FIV infection or throughout their lifespan. Thus, we were pleased to recognize that the cats were free of FcaGHV1, and therefore FcaGHV1 was not the underlying reason for the rather high frequency of lymphoma observed in our FeLV-infected SPF cats. However, we recognize that the samples from the SPF cats may not be representative to assess the contribution of FcaGHV1 to the development of lymphoma in field cats. 

## 5. Conclusions

In conclusion, this large-scale epidemiological study including cats from all Swiss cantons showed a molecular prevalence of 6.0%. Risk factors associated with FcaGHV1 detection were consistent with those identified in previous studies and included nonpedigree status and male sex. Adult cats (>3 years) were also at increased risk. Co-infection with other pathogens, such as hemoplasmas, FIV and presumptively progressive FeLV infection, was also a risk factor for FcaGHV1 detection. Although the molecular prevalence of FcGHV1 ranged from 0% to 16.7% in the different cantons, no clear influence of geographic or climatic factors or the assumed cat density was found. The clinical importance of FcaGHV1 is unclear, but the clinical information available only for a limited number of cats in the present study revealed FcaGHV1-positive cats with leukemia, carcinoma and cardiomyopathy and indicated that all 17 cats with lymphoma were FcaGHV1-negative. Further studies will be necessary to elucidate the pathogenic potential of FcaGHV1 infections in domestic cats.

## Figures and Tables

**Table 1:** Primers and probes employed in this study.

Primer Set	Name	Target Gene	Sequence (5′ - 3′)	Amplicon Size (bp^4^)	Reference
*Felis catus* gammaherpesvirus1 ^1^	Forward primer	Glycoprotein B	ACA TCT TCA CTG GAC AAC TGG	113	[20]
	Reverse primer		GTG CAT TTG ATG TCC TGA CTG		
	Probe		FAM - TGA ACA GCT GAG TCT CTA CAA GTC TCC A - BHQ1		
*Felis catus* gammaherpesvirus1 ^2^	Forward primer	Glycoprotein B	ACC TGC ACC AGA GCA TGA GA	360	[27]
	Reverse primer		TGT CCA GTA CGT TAG CCA ATC TTT		
Feline albumin ^3^	Forward primer	Albumin	GAT GGC TGA TTG CTG TGA TT	150	[51]
	Reverse primer		CCC AGG AAC CTC TGT TCA TT		
	Probe		FAM - ATC CCG GCT TCG GTC AGC - TAMRA		
Feline leukemia virus	Forward primer	FeLV LTR U3	AAC AGC AGA AGT TTC AAG GCC	131	[45]
	Reverse primer		TTA TAG CAG AAA GCG CGC G		
	Probe		FAM - CCA GCA GTC TCC AGG CTC CCC A - TAMRA		
Feline immunodeficiency virus	Forward primer	FIV gag	ATG GGG AAY GGA CAG GGG CGA GA	164	[54,55]
	Reverse primer		TCT GGT ATR TCA CCA GGT TCT CGT CCT GTA		
	Probe		FAM-TGG CCA TWA ARA (iQ500)GAT GYA GTA ATG TTG CTG TAG G-BHQ1		
*Mycoplasma haemofelis*	Forward primer	16S rRNA	GAA AGT CTG ATG GAG CAA TAC CAT	118	[53]
	Reverse primer		CTG GCA CAT AGT TWG CTG TCA CTT A		
	Probe		VIC - AGT ACT ATC ATA ATT ATC CCT CG - BHQ1		
‘*Candidatus* Mycoplasma haemominutum’	Forward primer	16S rRNA	GAA AGT CTG ATG GAG CAA TAC CAT	141	[53]
	Reverse primer		CTG GCA CAT AGT TWG CTG TCA CTT A		
	Probe		FAM - AAG GCT TAA TCA TTT CCT - BHQ1		
‘*Candidatus* Mycoplasma turicensis’	Forward primer	16S rRNA	GAA GGC CAG ACA GGT CGT AAA G	85	[52]
	Reverse primer		CTG GCA CAT AGT TWG CTG TCA CTT A		
	Probe		FAM - AAA TTT GAT GGT ACC CTC TGA - BHQ1		

^1^ Primers and probe used for the real-time PCR assay (113 bp amplicon size), ^2^ Primers used for sequencing (360 bp amplicon size), ^3^ The assay was used for quality control to test for the integrity of genomic DNA and the absence of PCR inhibitors; **^4^** bp, base pairs.

**Table 2:** Origin of the 881 samples included in the Swiss FcaGHV1 molecular prevalence study. The number of samples and the molecular FcaGHV1 prevalence rates are listed.

Canton of Switzerland	FcaGHV1-Negative	FcaGHV1-Positive	Total	% FcaGHV1 Prevalence (95% CI ^1^)
Aargau (AG)	46	5	51	9.8 (3.3–21.4)
Appenzell I.Rh. (AI)	21	3	24	**12.5** (2.7–32.4)
Appenzell A.Rh. (AR)	19	1	20	5.0 (0.1–24.9)
Bern (BE)	43	4	47	8.5 (2.4–20.4)
Basel-Landschaft (BL)	22	2	24	8.3 (1.0–27.0)
Basel-Stadt (BS)	19	2	21	9.5 (1.2–30.4)
Fribourg (FR)	42	3	45	6.7 (1.4–18.3)
Geneva (GE)	33	1	34	2.9 (0.1–15.3)
Glarus (GL)	20	0	20	0.0 (0–16.8)
Graubünden (GR)	37	1	38	2.6 (0.1-13.8)
Jura (JU)	20	4	24	**16.7** (4.7–37.4)
Lucerne (LU)	32	3	35	8.6 (1.8–23.1)
Neuchâtel (NE)	45	0	45	0.0 (0–7.9)
Nidwalden (NW)	36	4	40	10 (2.8–23.7)
Obwalden (OW)	29	2	31	6.5 (0.8–21.4)
St. Gallen (SG)	28	0	28	0.0 (0–12.3)
Schaffhausen (SH)	18	2	20	10.0 (1.2–31.7)
Solothurn (SO)	26	0	26	0.0 (0–13.2)
Schwyz (SZ)	43	4	47	8.5 (2.4–20.4)
Thurgau (TG)	22	0	22	0.0 (0–15.4)
Ticino (TI)	44	6	50	**12.0** (4.5–24.3)
Uri (UR)	19	1	20	5.0 (0.1–24.9)
Vaud (VD)	53	0	53	0.0 (0–6.7)
Valais (VS)	47	0	47	0.0 (0–7.5)
Zug (ZG)	22	2	24	8.3 (1.0–27.0)
Zurich (ZH)	42	3	45	6.7 (1.4–18.3)
Total	828	53	881	6.0 (4.5–7.8)

^1^ CI, confidence interval. The three highest FcaGHV1 molecular prevalences are marked in bold.

**Table 3:** Sample characteristics of the 881 cats in the Swiss prevalence study and the FcaGHV1 molecular prevalence rates.

Variable	Category	FcaGHV1-Negative (*n* = 828)	FcaGHV1-Positive (*n* = 53)	Total (*n* = 881)	% FcaGHV1 Prevalence (95% CI ^1^)
Pedigree	Yes	158	2	160	1.3 (0.2–4.4)
	No	440	39	479	8.1 (6.0–10.9)
	Unknown	230	12	242	5.0 (2.9–8.5)
Sex	M	53	3	56	5.4 (1.5–14.9)
	MC	278	30	308	9.7 (6.9–13.6)
	F	66	4	70	5.7 (2.2–13.8)
	FS	201	6	207	2.9 (1.3–6.2)
	Unknown	230	10	240	4.2 (2.3–7.5)
Age (years)	≤ 1	98	2	100	2.0 (0.4–7.0)
	>1 ≤ 2	50	0	50	0.0 (0.0–7.1)
	>2 ≤ 3	45	1	46	2.2 (0.1–11.3)
	> 3 ≤5	70	6	76	7.9 (3.7–16.2)
	> 5 ≤ 10	151	13	164	7.9 (4.7–13.1)
	> 10 ≤ 15	133	14	147	9.5 (5.8–15.4)
	> 15	47	6	53	11.3 (5.3–22.6)
	Unknown	234	11	245	4.5 (2.5–7.9)

^1^ CI, confidence interval; M, male; MC, male castrated; F, female; FS, female spayed.

**Table 4:** FcaGHV1 and feline leukemia virus (FeLV) infection in the 881 cats presented to Swiss veterinarians.

Variable	Category	FcaGHV1-Negative	FcaGHV1-Positive	% FcaGHV1 Prevalence (95% CI ^1^)	Odds Ratio (95% CI)	*P*-Value ^2^
FeLV	Aviremic	813	50	5.8 (4.3–7.6)	Ref.	
	Viremic	15	3	16.7 (3.6–41.4)	3.3 (0.97–11.1)	0.0884
FeLV provirus	Negative	785	49	5.2 (3.8–6.9)	Ref.	
	Positive	43	4	7.5 (2.1–18.2)	1.5 (0.6–5.5)	0.5202

^1^ CI, confidence interval; ^2^ Fisher’s exact test.

**Table 5:** Feline immunodeficiency virus (FIV) infection and feline hemoplasma infection in selected samples from the 881 cats presented to Swiss veterinarians.

Variable	Category	FcaGHV1-Negative	FcaGHV1-Positive	% FcaGHV1 Prevalence (95% CI ^1^)	Odds Ratio (95% CI)	*P*-Value ^2^
FIV ^3^	Negative	49	32	39.5 (28.8–51.0)	Ref.	
	Positive	3	20	87.0 (66.4–97.2)	**10.2** (2.8–37.2)	**<0.0001**
Hemoplasma ^4^	Negative	44	34	43.6 (32.4–55.3)	Ref.	
	Positive	9	19 ^5^	67.9 (47.6–84.1)	**2.7** (1.1–6.8)	**0.0463**

^1^ CI, confidence interval; ^2^ Fisher’s exact test; ^3^ FIV infection was investigated in 104 samples: 52 FcaGHV1-positive cats and 52 matched FcaGHV1-negative cats (see M&M). ^4^ Hemoplasma infections were investigated in 106 samples: 53 FcaGHV1-positive cats and 53 matched FcaGHV1-negative cats (see M&M). Hemoplasma-negative/-positive = negative for all hemoplasmas tested/positive for any of the three hemoplasmas tested. ^5^ This group included 3 cats co-infected with CMt and CMhm and one cat co-infected with Mhf and CMhm.

**Table 6:** Feline hemoplasma infections in the 91 stray cats from the canton of Jura (Switzerland).

Variable	Category	FcaGHV1-Negative	FcaGHV1-Positive	% FcaGHV1 Prevalence (95% CI ^1^)	Odds Ratio (95% CI)	*P*-Value ^2^
Hemoplasma ^3^	Negative	74	0	0.0 (0.0–4.9)	Ref.	
	Positive	12	5	29.4 (10.3–56.0)	**65.6** (3.4–1261)	**0.0001**

^1^ CI, confidence interval; ^2^ Fisher’s exact test; ^3^ Hemoplasma-negative/-positive = negative for all three hemoplasma species tested/positive for any of the three hemoplasmas tested.

**Table 7:** Clinical information for selected FcaGHV1-positive cats.

Cat ID	Breed	Age (Years)	Sex	Clinical Signs
2228157	EU-Longhair	7	MC	Hypertrophic cardiomyopathy, CKD
2156335	Maine Coon	13	FS	Bronchopneumonia
2235402	Sphinx	5	MC	Hypertrophic cardiomyopathy, pneumonia, pyothorax
2236869	EU-Shorthair	20	MC	CKD
2223639	EU-Shorthair	7	MC	Immune mediated hemolytic anemia, acute leukemia
2186282	EU-Shorthair	7	MC	Hypertrophic cardiomyopathy, chronic diarrhea
2200223	Maine Coon mixed breed	12	MC	Feline asthma, carcinoma (SCC)

ID, identity; EU, European cat; MC, male castrated; FS, female spayed; CKD, chronic kidney disease; SCC, squamous cell carcinoma.

## Supplementary Materials

The following are available online at https://www.mdpi.com/1999-4915/11/8/721/s1, Figure S1: Characteristics of the matched 53 FcaGHV1-negative cats selected for testing for other infections and comparison with the 53 FcaGHV1-positive cats.
