# Supplementary material for: Prevalence, Geographic Distribution, Risk Factors and Co-Infections of Feline Gammaherpesvirus Infections in Domestic Cats in Switzerland
Source: Viruses. 2019 Aug 6;11(8):721. doi: 10.3390/v11080721 (PMC6723517; doi:10.3390/v11080721)
Supplement: Supplementary file 1 [file viruses-11-00721-s001.pdf]

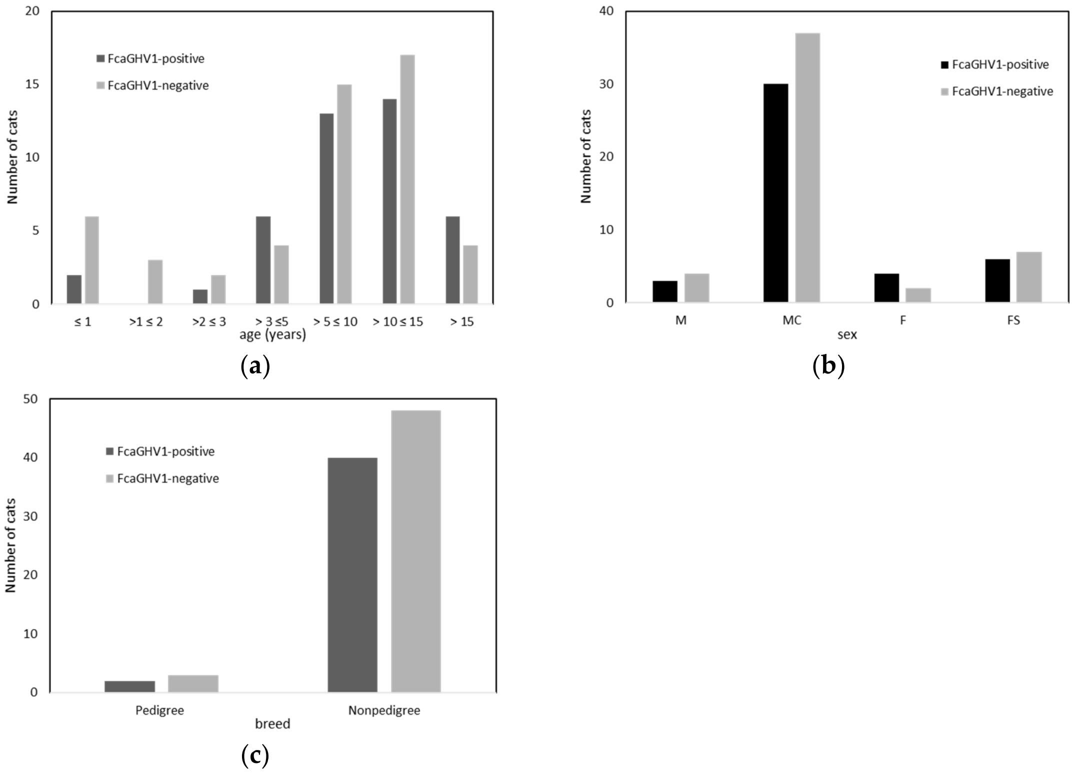

Figure S1. Characteristics of the matched 53 FcaGHV1-negative cats selected for testing for other infections and comparison with the 53 FcaGHV1-positive cats. The 106 tested samples of the 881 blood samples included 53 FcaGHV1 PCR-positive samples and 53 FcaGHV1 PCR-negative samples. The FcaGHV1 PCR-negative samples were matched to the FcaGHV1 PCR-positive samples based on the origin (Swiss canton of provenience), age (**a**), sex (**b**) and pedigree status (**c**) of the cat. There was no significant difference between FcaGHV1 PCR-negative and -positive cats regarding sex (pChi2 = 0.9171), percentage of pedigree cats (pChi2 = 1.000) and age (pChi2 = 0.0794). M, male; MC, male castrated; F, female; FS, female spayed.
